# A rapid culture independent methodology to quantitatively detect and identify common human bacterial pathogens associated with contaminated high purity water

**DOI:** 10.1186/s12896-015-0124-1

**Published:** 2015-02-18

**Authors:** Elizabeth Minogue, Nina L Tuite, Cindy J Smith, Kate Reddington, Thomas Barry

**Affiliations:** Nucleic Acid Diagnostics Research Laboratory (NADRL), Microbiology, School of Natural Sciences, National University of Ireland Galway, Galway, Ireland; Marine Microbial Ecology Laboratory, Microbiology, School of Natural Sciences, National University of Ireland Galway, Galway, Ireland

**Keywords:** Human microbial pathogens, Multiplex real-time PCR NAD assays, Biocontaminated HPW distribution systems, Biological water quality assurance

## Abstract

**Background:**

Water and High Purity Water (HPW) distribution systems can be contaminated with human pathogenic microorganisms. This biocontamination may pose a risk to human health as HPW is commonly used in the industrial, pharmaceutical and clinical sectors. Currently, routine microbiological testing of HPW is performed using slow and labour intensive traditional microbiological based techniques. There is a need to develop a rapid culture independent methodology to quantitatively detect and identify biocontamination associated with HPW.

**Results:**

A novel internally controlled 5-plex real-time PCR Nucleic Acid Diagnostics assay (NAD), was designed and optimised in accordance with Minimum Information for Publication of Quantitative Real-Time PCR Experiments guidelines, to rapidly detect, identify and quantify the human pathogenic bacteria *Stenotrophomonas maltophilia*, *Burkholderia* species, *Pseudomonas aeruginosa* and *Serratia marcescens* which are commonly associated with the biocontamination of water and water distribution systems. The specificity of the 5-plex assay was tested against genomic DNA isolated from a panel of 95 microorganisms with no cross reactivity observed. The analytical sensitivities of the *S. maltophilia*, *B. cepacia, P. aeruginosa* and the *S. marcescens* assays are 8.5, 5.7, 3.2 and 7.4 genome equivalents respectively.

Subsequently, an analysis of HPW supplied by a Millipore Elix 35 water purification unit performed using standard microbiological methods revealed high levels of naturally occurring microbiological contamination. Five litre water samples from this HPW delivery system were also filtered and genomic DNA was purified directly from these filters. These DNA samples were then tested using the developed multiplex real-time PCR NAD assay and despite the high background microbiological contamination observed, both *S. maltophilia* and *Burkholderia* species were quantitatively detected and identified. At both sampling points the levels of both *S. maltophilia* and *Burkholderia* species present was above the threshold of 10 cfu/100 ml recommended by both EU and US guidelines.

**Conclusions:**

The novel culture independent methodology described in this study allows for rapid (<5 h), quantitative detection and identification of these four human pathogens from biocontaminated water and HPW distribution systems. We propose that the described NAD assay and associated methodology could be applied to routine testing of water and HPW distribution systems to assure microbiological safety and high water quality standards.

**Electronic supplementary material:**

The online version of this article (doi:10.1186/s12896-015-0124-1) contains supplementary material, which is available to authorized users.

## Background

Maintaining high water quality standards is crucial, as water is essential to human health, development and well-being. The efficient operation and biological control of water distribution systems is important as High Purity Water (HPW), municipal and recreational water contaminated with waterborne pathogens such as non-fastidious Gram-negative bacteria non *Enterobacteriaceae* (GNB-NE) can pose a serious risk to human health. Water, and in particular HPW, is a vital component of both the clinical and many industrial manufacturing sectors such as the pharmaceutical, food and beverage and semiconductor industries [1-3].

In healthcare facilities, such as neonatal units, intensive care units, dental practices and clinical diagnostics testing laboratories, water and HPW is routinely used for hand washing, bathing, cleaning semi-critical areas washing and rinsing patient medical devices and in the preparation and processing of medicines and other health products [4,5]. A recent traditional culture based microbiological study of water and water distribution systems in a number of hospital departments revealed high levels of contamination by GNB-NE [6]. In such settings, this microbial contamination with opportunistic pathogens could result in the transmission of Healthcare Associated Infections (HCAIs) in immunocompromised patient groups [7-10]. There have been many reported cases of HCAIs associated with microbial contamination of water and HPW delivery systems and exposure to these water-borne pathogens in hospitals can result in increased patient morbidity and mortality [11-13]. It has also been observed that the use of biocontaminated water and HPW for clinical culture based microbiological diagnostics can result in the outbreak of pseudoepidemics [14,15]. Such clinical derived pseudoepidemics can result in costly additional and unnecessary diagnostic procedures and therapeutic interventions for patients [16-19].

In industrial settings, microbial contamination of water and HPW distribution systems can result in the biodeterioration of manufacturing equipment and as previous studies have demonstrated contamination with microorganisms can severely compromise the quality of industrial raw materials and finished products [20-22].

Previous studies have demonstrated that the microbial load present in water distribution systems can be significantly underestimated using the traditional plate count microbial culture method as a result of the presence of physiologically active bacteria that are unable to form colonies on culture media [23,24]. These viable but non-culturable bacteria may therefore be undetectable by standard microbial cultivation [25]. It has also been observed that variables such as the sampling technique and culture medium used can be crucial in obtaining accurate and reproducible results when testing these water samples [26]. Such studies demonstrate the need for and importance of real-time monitoring for the onset of biocontamination of HPW purification units and HPW delivery systems by rapid microbial culture independent methods such that prompt remedial action may be undertaken.

Therefore, there is a need to develop rapid, sensitive and high throughput methodologies for the quantitative detection and identification of biocontamination associated with HPW to ensure appropriate standards and quality can be achieved. Nucleic Acid Diagnostics (NAD) technologies offer the potential to analyse in real-time the onset and progress of microbial biocontamination associated with HPW distribution systems. Previously, we have reported the design and development of an internally controlled multiplex real-time PCR NAD assay for both *P. aeruginosa* and *Burkholderia* species for use in the quantitative detection and identification of these GNB-NE in biocontaminated HPW under laboratory conditions [27]. For the present study, additional novel NAD targets for two more GNB-NE, *S. maltophilia and S. marcescens* have been incorporated resulting in a 5-plex real-time PCR NAD assay, inclusive of an Internal Amplification Control (IAC).

This study which was designed in accordance with Minimum Information for Publication of Quantitative Real-Time PCR Experiments (MIQE) guidelines [28] describes a methodology combining water filtration, DNA purification and NAD technologies to detect, identify and absolutely quantify these common human pathogenic contaminates of HPW. Furthermore, we have also demonstrated the developed methodology by testing a HPW delivery system supplied by a Millipore Elix 35 water purification unit distributing HPW based in a public building facility. The Millipore Elix 35 system uses both reverse osmosis and electrodeionization for production of HPW. Using the developed methodology, we determined that the HPW delivery system was contaminated with the human pathogens, *S. maltophilia* and *Burkholderia* species.

## Methods

### Bacterial strains, microbial culture media and growth conditions

Ten *Stenotrophomonas* species/strains, 10 *Burkholderia* species, 26 *Pseudomonas* species/strains and 10 *Serratia* species/strains were used in this study (Additional file 1: Table S1). All target species/strains were purchased from either the German Collection of Microorganisms and Cell Cultures (Deutsche Sammlung von Mikroorganismen und Zellkulturen GmbH, DSMZ. Braunschweig, Germany) or from BCCM Belgian Co-ordinated Collections of Microorganisms bacterial collection (LMG, Gent, Belgium) (Additional file 1: Table S1).

All *Stenotrophomonas*, *Burkholderia*, *Pseudomonas* and *Serratia* species were grown in tryptone soya broth (Oxoid, Cambridge, UK) or R2A broth (Lab M limited, Lancashire, United Kingdom) at either 30°C or 37°C for approximately 12 - 24 h or until sufficient growth was observed. For all other microorganisms tested in the study, total genomic DNA was provided from microbial stocks held within this laboratory (Additional file 1: Table S1).

### DNA isolation and quantification

Total genomic DNA was isolated from overnight broth culture using the Quick-gDNA MiniPrep kit (Zymo Research, California, USA). Briefly, 1.5 ml of culture was centrifuged in a bench-top centrifuge at 18,000 g for two min. The supernatant was discarded and the pellet was resuspended in 400 μl Genomic Lysis Buffer by vortexing briefly, then incubated at room temperature for 5-10 min. Subsequently, steps 2-5 of the purification of total DNA from cell suspensions and proteinase K digested samples procedure were followed according to the manufacturer’s guidelines (http://www.zymoresearch.com/downloads/dl/file/id/18/d3006i.pdf).

DNA integrity was assessed on a 1% w/v agarose gel. Concentrations for all total genomic DNA used in this study were determined using the Quant-iT™ dsDNA HS Assay Kit and the Qubit fluorometer (Invitrogen Corporation, California, USA). All DNA samples were stored at -20°C prior to use.

### Diagnostics target identification and development

The diagnostics target genes used in this study were identified by *in silico* evaluation of a number of genes currently under investigation and evaluation in this laboratory for use in nucleic acid based diagnostics test systems. These include the *ssrA*, *lepA*, *rnpB*, 16S rRNA, and genes and the 16 – 23 S rRNA ITS. These gene targets have all previously been reported as useful species specific diagnostics targets for use in nucleic acid based diagnostics [29-35]. Nucleotide sequence information was retrieved from the National Center for Biotechnology Information (NCBI) website (http://www.ncbi.nlm.nih.gov), the Functional Gene Pipeline and Repository website (http://fungene.cme.msu.edu), the RNaseP database (http://www.mbio.ncsu.edu/rnasep/) and the tmRNA databse (http://www.ag.auburn.edu/mirror/tmRDB/). Alignments of these nucleotide sequences were carried out using the clustalW2 multiple sequence alignment programme (http://www.ebi.ac.uk/Tools/clustalw2/index.html).

### Conventional and real-time PCR primers and Taqman hydrolysis probe design

All oligonucleotide primers and hydrolysis probes were designed manually according to recommended general guidelines [28,36]. Nucleotide sequencing primers were designed to have a melting temperature (Tm) of between 60-62°C. The oligonucleotide primers used for real-time PCR were designed to have a Tm of 58-61°C, with all probes designed to have a Tm 6-8°C higher (Table 1). To determine the specificity of each assay designed *in silico* all primers and probes were BLAST analysed. Subsequently, the performance characteristics of each primer set was analysed in the laboratory along with the specific probe. The most robust assay combination, as reflected by PCR efficiency, which had an LoD of < 10 GE was used in the final assay. Oligonucleotide primers and probes were supplied by *Eurofins* MWG Operon (Ebensburg, Germany), TIB MOLBIOL (Berlin, Germany) or Integrated DNA Technology (Leuven, Belgium).

**Table 1:** Oligonucleotide primers and probes used in this study

**Name**	**Function**	**Sequence (5′-3′)**	**Target gene**
CIAC F	Forward composite primer for IAC generation	TGAGGAAGACTTATTGGCTGATACCCAACTTGGAATG	Chimeric PCR product
CIAC R	Reverse composite primer for IAC generation	CGGTACATTGTGGTCTTTAAGTCTTCACCAGAATAAAATTG	Chimeric PCR product
Ser F	Sequencing forward primer	GTGGAGCTGGGGGGAT	*ssrA*
Ser R	Sequencing forward primer	GGGGCTGATTCTGGATTCG	*ssrA*
Psd F1	Sequencing forward primer	GGATTTGAACCCCCGTCC	*ssrA*
Psd R2	Sequencing reverse primer	AGGATTCGACGCCGGT	*ssrA*
BF1	Sequencing forward primer	GAGGAAAGTCCGGACTCC	*rnpB*
BF2	Sequencing forward primer	GGCAGGGTGATGGCTAA	*rnpB*
BR2	Sequencing reverse primer	GATAAGCCGGATTCTGTGC	*rnpB*
Sten F	Sequencing forward primer	GGGGMYTACGGWTTCGAC	*ssrA*
Sten R	Sequencing forward primer	GGGARTCGAACCRSGTCC	*ssrA*
IAC F	IAC forward primer	TGAGGAAGACTTATTGGCTG	Chimeric PCR product
IAC R	IAC reverse primer	CGGTACATTGTGGTCTTTAAG	Chimeric PCR product
IAC P	IAC specific hydrolysis probe	TYE665-TCCTTAGATGGTACCAGTGCCA- IBRQSp	Chimeric PCR product
S. mar F	*S. marcescens* forward primer	GCACTACATGCTTAGTCCAAT	*ssrA*
S. mar R	*S. marcescens* reverse primer	CAGCTTAATACCCTGCTTAGAG	*ssrA*
S. mar P	*S. marcescens* specific hydrolysis probe	ROX-CACGCTACTGACAGACTAGCCT-BHQ2	*ssrA*
BF1	*Burkholderia* forward primer	GAGGAAAGTCCGGACTCC	*rnpB*
BR1	*Burkholderia* reverse primer	TCTTACCGCACCGTTTCA	*rnpB*
Burk P	*Burkholderia* genus specific hydrolysis probe	FAM-ACACGCGGAACAGGGCAA-BHQ1	*rnpB*
PF2	*P. aeruginosa* forward primer	GACAGTCGTTTCGGGTTTAC	*ssrA*
PR1	*P. aeruginosa* reverse primer	CGACGACAACTACGCTCT	*ssrA*
P. aer P	*P. aeruginosa* specific hydrolysis probe	HEX-GCATCCCCTAGCGACTGCT-BHQ1	*ssrA*
S. malt F	*S. maltophilia* forward primer	TACATGCTTAGCTCACCGT	*ssrA*
S. malt R	*S. maltophilia* reverse primer	CGAAACTGCTTGTGTCCAT	*ssrA*
S. malt P	*S. maltophilia* specific hydrolysis probe	CYAN500-CTGTTGTGTTCTAGTGCCGGAC-BHQ1	*ssrA*

### Conventional PCR for sequencing

Conventional PCR was performed on the iCycler iQ thermal cycler (Bio-Rad Laboratories Inc., California, USA). PCR reactions were carried out in 0.2 ml PCR tubes, containing forward and reverse primer (0.2 μM final conc.), 2.5 μl 10X buffer (15 mM MgCl_2_), 0.5 μl dNTP mix (10 mM: deoxynucleoside triphosphate set, 0.5 U Taq DNA polymerase Roche Diagnostics, Basel, Switzerland), 1 μl of template DNA and adjusted to a final volume of 25 μl with the addition of nuclease-free water (Applied Biosystems, California, USA). The thermal cycling parameters consisted of an initial denaturation step at 95°C for 4 min, followed by 35 cycles of denaturation at 95°C (30 s), amplification at 50°C (30 s), and extension at 72°C (30 s), followed by a final elongation at 72°C for 7 min. Sequencing primers are outlined in Table 1.

### Nucleic acid diagnostics target sequencing

Nucleotide sequence data used for real-time PCR NAD assay design and development was obtained either from publicly available databases or were generated in this study. Sequencing primers (Table 1) were designed to amplify the *ssrA* gene of *Stenotrophomonas*, and *Serratia* species. Amplification of the *ssrA* gene of *P. aeruginosa* and the *rnpB* gene of *Burkholderia* species was performed as previously described [27]. Conventional PCR products were generated as described above, followed by purification using the High Pure PCR product purification kit (Roche Diagnostics). The purified PCR products were sequenced externally (Sequiserve, Vaterstetten, Germany).

### Internal Amplification Control for multiplex real-time PCR NAD assay

The IAC used in this multiplex real-time diagnostics assay as previously described [27] was developed using a modified version of the composite primer approach [37-39]. Briefly, the IAC amplifies and detects a chimeric PCR product containing nucleotide sequence of both *Candida albicans ALS1* gene and the *aqualysin 1* gene from *Thermus aquaticus.*

### Real-time PCR

Real-time PCR NAD assays were initially tested in a monoplex format to determine the specificity and analytical sensitivity. Monoplex real-time PCR reactions were performed on the LightCycler 2.0 instrument using the LightCycler TaqMan Master kit (Roche Diagnostics). Each monoplex reaction contained 5X master mix (4 μl), forward and reverse primer (0.5 μM final conc.), probe (0.2 μM final conc.), 0.5 U uracil-DNA glycosylase (UDG,New England Biolabs), 5% v/v Dimethyl sulphoxide (DMSO), template DNA (2 μl) and the volume adjusted to 20 μl with the addition of nuclease-free water. The thermal cycling parameters consisted of 10 min at 37°C to incubate UDG followed by 10 min incubation at 95°C to deactivate UDG, activate the polymerase and denature the template DNA. Subsequently, 50 cycles of 95°C for 10 s and 60°C for 30 s were performed, with a final a cooling step at 40°C for 10 s. The temperature transition rate for all cycling steps was 20°C/s.

For multiplex real-time PCR the *S. maltophilia, Burkholderia* species and *P. aeruginosa* specific probes were labelled with Cyan 500, FAM, HEX and Black Hole Quencher 1 (BHQ1) respectively. The *S. marcescens* probe was labelled with ROX and Black Hole Quencher 2 (BHQ2). The IAC assay probe was labelled with TYE665 and Iowa Black® RQ-Sp (IBRQSp). The multiplex real-time PCR NAD assay was optimised to the required standards routinely used in this laboratory and the specificity and analytical sensitivity was determined.

Multiplex real-time PCR reactions were subsequently performed using the LightCycler 480 probes master kit (Roche Diagnostics) on the LightCycler 480 System I. Each multiplex reaction contained 2 X master mix (10 μl), forward and reverse primers (0.5 μM final conc.) CYAN500, FAM, HEX, ROX and TYE665 probes (0.2 μM final conc.), UDG (0.4 U), DMSO (5% v/v). Template DNA (5 μl) and IAC DNA (1×10^4^ recombinant plasmid copies) were added to each reaction mix. Nuclease-free water was added to a final volume of 20 μl. A no template control reaction was included in each experiment. The cycling parameters used for multiplex reactions were in accordance with monoplex parameters (see previous paragraph). The temperature transition rate, referred to as the ramp rate on the LightCycler 480, was 4.4°C/s while heating and 2.2°C/s on cooling. As outlined by the manufacturer [40] a colour compensation file was generated prior to experimental analysis to avoid fluorescence leakage between channels.

### Determining the specificity of the multiplex real-time diagnostic assay

To demonstrate the specificity of the designed multiplex real-time PCR NAD assay, it was tested against genomic DNA isolated from a panel of 95 microorganisms (Additional file 1: Table S1) at a concentration of 10^4^ Genome Equivalents (GE) per reaction. Genome sizes were available from the NCBI website for all microorganisms of interest and were used to calculate the GE of each microorganism tested using previously published guidelines [41].

### Analytical sensitivity of the multiplex real-time diagnostic assay determined using probit regression analysis

The analytical sensitivity of each assay in multiplex format was established by testing twelve replicates of eight bacterial genomic DNA concentrations, using probit regression analysis [42]. To determine the impact of genomic DNA from other microorganisms commonly associated with biocontaminated HPW distribution systems the LOD was also investigated in the presence of a background of pooled genomic DNA from 10 other bacteria commonly associated with water system contamination [24,43,44]. The final total concentration of the pooled bacterial genomic DNA was 10^4^ GE per reaction. These bacteria were *Klebsiella pneumonia* DSM 9377, *Ralstonia sp.* DSM 13640, *Enterobacter aerogenes* NCTC 10006, *Proteus mirabilis* DSM 4479, *Staphylococcus aureus* DSM 21705, *Acinetobacter baumannii* DSM 30007, *Bacillus cereus* DSM 31 and 3 out of 4 of the following *S. maltophilia* DSM 50170, *B. cepacia* DSM 7288, *P. aeruginosa* DSM 19880 and *S. marcescens* DSM 30121 (genomic DNA from bacterial target subject to analytical sensitivity testing not included in background genomic DNA). The IAC, at a concentration of 10^4^ plasmid equivalents per reaction, was also included in all samples during sensitivity testing.

### Generation of external standard curves

Three overnight microbial cultures of either *S. maltophilia*, *Burkholderia* species (for standard curve generation *B. cepacia* DSM 7288 the type strain of the type species of the *Burkholderia* genus was used), *P. aeruginosa* or *S. marcescens* grown in R2A broth with an optical density at 600 nm (OD_600_) of 0.1 were serially diluted tenfold (6 dilutions) in sterile phosphate buffered saline (PBS) (Oxoid). One hundred μl of each dilution were spread on R2A agar and incubated for 48 h at 30°C. These plates were used to count cfu. DNA was isolated from two hundred μl aliquots from each dilution (in triplicate) using the Quick-gDNA MiniPrep kit (Zymo Research) as per protocol outlined above. DNA was eluted in 50 μl sterile nuclease free water which was then used as template DNA in the multiplex real time PCR reaction. Standard curves were derived by plotting threshold cycle (C_T_) values against overnight plate counts (CFU (log_10_)).

### Sampling from HPW delivery system for microbiological analysis

An Elix 35 HPW purification unit (Merck Millipore, Massachusetts, USA) and HPW delivery system based in a public building facility was investigated for bacterial contamination. The last full service of the Elix 35 HPW purification unit was carried out approximately 36 months prior to first sampling. Sampling of HPW was performed at two point of use sources. Sampling point A was an outlet valve adjacent to the Elix 35 HPW purification unit and point B was a laboratory tap approximately 25 - 30 m from the Elix 35 HPW purification unit. During the course of this study the Elix 35 HPW purification unit had a new Progard TL1 System Cl2 Autoclean Pretreatment Pack and the Q-Gard TL Polishing Pack fitted. Samples of the HPW were subsequently taken from both points after this process was carried out. Before taking HPW samples, the outlets exteriors were cleaned with 70% ethanol, and HPW was subsequently allowed to flow for 2 min (~300 ml/min). Five litre samples were collected into sterile containers and filtered using 0.45 μm, 47 mm cellulose nitrate/acetate membrane filters (Millipore SAS, Molsheim Cedex, France) [45]. Each filter was placed in 1500 μl Genomic Lysis Buffer (Quick-gDNA MiniPrep kit, Zymo Research) and incubated on rotating platform at room temperature for 5 - 10 min followed by steps 2 - 5 of the total DNA isolation from cell suspension procedure according to the manufacturer’s guidelines. The DNA was eluted in 50 μl sterile nuclease free water.

### Microbiological analysis of HPW delivery system

(i) Conventional culture methods for the microbiological analysis of HPW delivery system.

Heterotrophic Plate Counts (HPCs) were performed by plating 100 μl of HPW onto R2A agar and incubating at 37°C for 48 h. Additionally, 100 μl samples of HPW were plated on four selective agars. These were *Pseudomonas* agar, L-arabinose ornithine irgasan medium [46], *B. cepacia* medium, and *B. cepacia* specific agar (Fannin, Ireland). (Growth of *S. maltophilia* on *B. cepacia* medium was confirmed by culture of *S. maltophilia* strains (DSM 50170^T^ and DSM 50173) at 30°C for 24 h). Plates were incubated at 30°C for 48 h. Single colonies were isolated and these microbial cultures were used to inoculate the BBL Crystal Enteric/Nonfermenter ID system panels (Becton Dickinson, New Jersey, USA), which were incubated at 37°C for 20 h. Results were evaluated using BBL Crystal ID System Electronic Codebook.

(ii) Rapid NAD quantitative detection and identification of bacteria from a biocontaminated HPW delivery system.

Total genomic DNA isolated from filtered HPW samples was used as template DNA in the developed multiplex real-time PCR NAD assay to determine if the HPW samples were contaminated with *S. maltophilia*, *Burkholderia* species, *P. aeruginosa* or *S. marcescens*. In order to determine the degree of contamination of the HPW delivery system with these bacteria each positive result was absolutely quantified by comparison to one of the established external standard curves generated as described in section Generation of external standard curves.

## Results

### Diagnostics target identification and development

The NAD targets used in this study for the specific detection of the *Burkholderia* species and *P. aeruginosa* were previously described [27]. For the specific detection of the pathogens *S. maltophilia* and *S. marcescens in silico* analysis of a number of genes for use as a nucleic acid target was carried out (data not shown). The usefulness of these NAD target genes (*ssrA*, *lepA*, *rnpB*) has been previously demonstrated with the development of NAD assays to specifically detect microorganisms of interest [29-34,47]. The analysis of the *ssrA* gene revealed significant intragenic nucleotide sequence variation between closely related species within the nucleotide gene sequences of *S. maltophilia* and *S. marcescens* to allow for the design of species specific probes for both these microorganisms of interest. A BLAST analysis of each target probe did not show any cross reactivity between specific probes and any other non target species/strains (data not shown).

### Specificity of the multiplex real-time diagnostic assay

The specificity of each real-time PCR NAD assay was confirmed both in monoplex and multiplex formats using the panel of microorganisms listed in Additional file 1: Table S1. The *S. maltophilia* and *S. marcescens* specific real-time PCR NAD assays each detected five positive control strains and did not detect any of the remaining 90 microorganisms tested in this study. The *P. aeruginosa* specific real-time PCR NAD assay detected the 14 *P. aeruginosa* strains in our collection. The remaining 81 microorganisms tested were not detected. Ten *Burkholderia* species were tested using the *Burkholderia* genus specific real-time PCR NAD assay. All 10 *Burkholderia* species were detected and none of the remaining 85 microorganisms tested were detected. The specificity of the IAC assay was also tested using the same panel of microorganisms and no cross reactivity was observed. For complete results of specificity testing see Table 2.

**Table 2:** Microbial specificity testing of the developed multiplex assay

**Assay**	**Diagnostics gene target**	**Inclusivity testing**	**Exclusivity testing**
*S. maltophilia*	*ssrA*	5/5	0/90
*Burkholderia* genus	*rnpB*	10/10	0/85
*P. aeruginosa*	*ssrA*	14/14	0/81
*S. marcescens*	*ssrA*	5/5	0/90

### Analytical sensitivity testing of the multiplex real-time PCR NAD assay

The LOD of each diagnostics assay was evaluated in both monoplex and multiplex real-time PCR formats. Total genomic or plasmid DNA was quantified and serial dilutions were prepared from 10^5^ to 0.1 genome/plasmid equivalents. The dilution series was run in triplicate for each monoplex real-time PCR NAD assay. In a monoplex format, all five diagnostics assays had an analytical sensitivity of less than 10 GE. The LOD of the real-time multiplex diagnostics assay was established using probit regression analysis with 12 replicates for each dilution. With 95% confidence, the analytical sensitivities of the *S. maltophilia*, *B. cepacia, P. aeruginosa* and the *S. marcescens* assays are 8.5 GE, 5.7 GE, 3.2 GE and 7.4 GE respectively. The LOD for each assay when investigated in the presence of a background of pooled genomic DNA from 10 other microorganisms at a concentration of 10^4^ GE per reaction are 9.5 GE, 6.5 GE, 3.3 GE and 7.7 GE respectively. The IAC, at a concentration of 10^4^ plasmid equivalents per reaction, was included in all samples during sensitivity testing and was detected at C_T_ values from 27.2 – 28.6. The amplification efficiency (E) of the multiplex real-time PCR reactions ranged from 94 – 102%.

### Limit of quantification (LOQ)

DNA extracted from different concentrations of overnight culture was used as template DNA in the multiplex real-time PCR reaction. Standard curves were derived by plotting threshold cycle (C_T_) values against overnight plate counts (CFU (log_10_)). The dynamic range of each assay was established and the limit of quantification for each assay was determined. The LOQ of the *S. maltophilia*, *B. cepacia, P. aeruginosa* and the *S. marcescens* assays are 88.5 cfu, 59.7 cfu, 73.2 cfu and 87.4 cfu respectively.

### Microbiological analysis of a HPW delivery system

(i) Conventional culture methods for the microbial analysis of a HPW delivery system.

HPW samples were cultured on R2A agar to determine HPCs. HPCs observed in the HPW delivery system ranged from 2.93 × 10^6^ – 3.67 × 10^6^ / L (Additional file 1: Table S2). This demonstrates naturally occurring high bacterial contamination in the HPW delivery system. A biochemical analysis of microbial colonies isolated on R2A agar and selective agar was performed using the BBL crystal enteric NF system. This analysis confirmed the presence of both *S. maltophilia* and *Burkholderia* species in the HPW delivery system. However, neither *P. aeruginosa* nor *S. marcescens* were detected using this analysis. Other bacteria identified in the HPW delivery system using the BBL crystal enteric NF system were *Sphingomonas* sp., *Flavinonas* sp and *Vibrio* sp. The Turn Around Time (TAT) to result for the microbiological analysis of the HPW delivery system using conventional microbial culture methods was > 72 h.

(ii) Rapid NAD quantitative detection and identification of bacteria in a HPW delivery system.

The dynamic range for the external standard curves used in this study for the absolute quantification of pathogenic bacteria present in HPW delivery system was 10^2^-10^7^ cfu. For the quantitative detection and identification of bacteria in the HPW delivery system total genomic DNA was extracted from each filter and used as a template in the multiplex real-time PCR NAD assay developed in this study. The results were absolutely quantified by comparison to the external standards curves (see section Generation of external standard curves). From this analysis, it was determined that the HPW delivery system was contaminated with both *S. maltophilia* and *Burkholderia* species. The concentration of *S. maltophilia* detected in the HPW ranged from 5.75 × 10^1^ - 7.65 × 10^1^ cfu/100 ml at sampling point A and 1.30 × 10^2^ – 1.72 × 10^2^ cfu/100 ml at sampling point B. The concentration of *Burkholderia* species observed in the HPW was higher with concentrations of 4.06 ×10^2^- 7.42 × 10^2^ cfu/100 ml at sampling point A and 6.88 × 10^2^ – 1.17 × 10^3^ cfu/100 ml at sampling point B. *P. aeruginosa* and *S. marcescens* were not detected. Both European and US guidelines for microbial analysis of HPW recommend that under normal conditions an appropriate action level is a microbial count of 10 cfu/100 ml [48,49]. The concentration of both *S. maltophilia* and *Burkholderia* species detected in the HPW distribution system tested was above the recommended action level. For complete results of this analysis see Table 3. The TAT to result for the microbiological analysis of the HPW delivery system using the multiplex real-time PCR NAD assay was < 5 h.

**Table 3:** Microbiological analysis of a HPW delivery system using developed methodology

**Sampling point**	**Sampling time**	**C**_**T**_ **value (SD)**	^**a**^ **Mean calculated cfu/100 ml (SD)**	**C**_**T**_ **value (SD)**	^**a**^ **Mean calculated cfu/100 ml (SD)**	**Above threshold EU/US guidelines action level of > 10 cfu/100 ml**
		***S. maltophilia***	***S. maltophilia***	***Burkholderia***	***Burkholderia***	
A	1	29.02 (0.07)	7.7 × 10^1^ (37)	27.14 (0.15)	7.4 × 10^2^ (145)	Yes
A	2	29.25 (0.21)	6.5 × 10^1^ (103)	27.81 (0.23)	6.2 × 10^2^ (97)	Yes
A	3	29.40 (0.04)	5.8 × 10^1^ (13)	28.47 (0.22)	4.1 × 10^2^ (560)	Yes
B	1	27.90 (0.14)	1.7 × 10^2^ (162)	27.64 (0.07)	6.9 × 10^2^ (333)	Yes
B	2	28.30 (0.27)	1.3 × 10^2^ (246)	27.16 (0.03)	9.4 × 10^2^ (167)	Yes
B	3	28.10 (0.12)	1.5 × 10^2^ (139)	26.83 (0.16)	1.2 × 10^3^ (867)	Yes

C_T_ Threshold cycle.

^a^Mean calculated cfu/100 ml calculated by multiplex real time PCR.

A Valve adjacent to Elix 35 HPW purification system.

B Laboratory tap approximately 25 - 30 m from the Elix 35 HPW purification system.

^1^Before Pretreatment and Polishing Pack change.

^2^one day post Pretreatment and Polishing Pack change.

^3^one week post Pretreatment and Polishing Pack change.

## Discussion

The use of rapid quantitative multiplex real-time PCR NAD assays for the analysis of microbial contamination in water and HPW distribution systems can potentially overcome some of the limitations that are associated with conventional culture based microbiological diagnostics techniques traditionally used to monitor water quality. Conventional microbial analysis of water and HPW distribution systems by traditional culture techniques are labour-intensive and can take between 2 - 7 days, which can result in significant delays in identifying the contaminating microorganism [50]. Furthermore, conventional microbial culture methodologies traditionally used to analyse the biocontamination of water and HPW distribution systems assess the number of bacteria that can form visible colonies on a solid medium under specific test growth conditions (e.g. incubation temperature, culture medium and incubation time). However, these approaches only reveal the presence of bacteria culturable under these conditions and may not grow all the contaminating microorganisms present [51]. Therefore, monitoring water quality by HPCs enumeration with conventional microbial culture methods may only represent a relatively small proportion of the total viable microbial population in a biocontaminated water or HPW distribution system [25].

It is possible that the use of NAD technologies such as real-time PCR for the analysis of microbial contamination in water and HPW distribution systems could result in the detection of DNA from dead microorganisms. This may result in an overestimation of the microbial contamination or the risk of false positive results. However, the presence of any DNA from pathogenic microorganisms in a water sample indicates that there is biological contamination within the system. Any contamination with pathogenic microorganisms within water distribution systems even if present at low concentrations should be considered a risk.

In a healthcare setting, if the water or HPW distribution system is suspected as a potential source of HCAIs, the ability to specifically detect and identify pathogenic microorganisms rapidly would allow the required infection control and prevention measures to be taken promptly. A delay in this analysis could result in an increased risk of patients contracting HCAIs and subsequently a significant increase in both the duration and cost of a healthcare stay. In this setting, where there is an increasing proportion of immunologically compromised patients there is added potential for infection by pathogenic microorganisms originating from biocontaminated water and HPW distribution systems. A recent commentary on waterborne pathogen detection [52] highlights that although hospital water and HPW distribution systems have been implicated in the spread of HCAIs there is a lack of evidence of direct transmission from water distribution systems. However, it concludes that a microbiological investigation of the hospital water distribution system, such as that performed by Cristina *et al.* [6], may be beneficial in identifying the position, frequency and extent of contamination. Such analysis could then be used to assess the degree of risk to patients. Therefore an ability to specifically detect and identify pathogenic microorganisms in the HPW distribution system rapidly may aid in determining the potential source and causative agent of the HCAIs, thereby allowing the required infection control and prevention measures to be taken promptly.

In this current study the internally controlled novel multiplex real-time PCR diagnostics assay developed uses NAD targets for the rapid specific quantitative detection and identification of four important human pathogenic bacteria associated with the contamination of HPW distribution systems [24,43,53]. The developed multiplex real-time PCR NAD assay was determined to be 100% specific when tested against a panel of 95 microorganisms with no cross reactivity observed (Table 2 and Additional file 1: Table S1). To our knowledge, this is the first report of an internally controlled 5-plex assay for the quantitative detection and identification of *S. maltophilia*, *Burkholderia* species, *P. aeruginosa* and *S. marcescens* for use in the microbial analysis of a HPW distribution system.

The analytical sensitivity of all four diagnostics assays described in this study is less than 10 GE allowing for the potential detection of low levels of pathogenic bacteria present in a biocontaminated HPW distribution system. The 5-plex PCR NAD assay described does not require microbial culture pre-enrichment of the HPW sample to increase analytical sensitivity prior to running the diagnostics assay. This ability to detect contaminating microorganisms without microbial culture pre-enrichment is a significant advantage, as the inclusion of a microbial culture pre-enrichment step eliminates the ability to absolutely quantify the contaminating microorganism in the HPW. This is important as routine testing of HPW needs to be both qualitative and quantitative [48]. Another major advantage of not including a pre-incubation step is that the assay TAT is significantly reduced. Using the developed multiplex real-time PCR NAD assay, the TAT to result inclusive of HPW sampling and filtration, DNA preparation, real-time PCR reaction, and data analysis is < 5 h in contrast to 2-7 days using traditional microbial culture diagnostics methodologies [49].

A HPW delivery system supplied by an Elix 35 HPW purification unit was evaluated for microbiological contamination using both traditional microbial culture methodologies and the developed NAD assay and associated methodology. This analysis was performed both before and after a pretreatment and polishing pack change of the Millipore Elix 35 HPW purification unit. The developed multiplex real-time PCR NAD assay determined that the HPW delivery system was contaminated with the opportunistic human pathogens *S. maltophilia* and *Burkholderia* species. The numbers of both of these GNB-NE present in the HPW distribution system was above the action level threshold of 10 cfu/100 ml recommended by both European and US guidelines [48,49]. The presence of both *S. maltophilia* and *Burkholderia* species in the HPW delivery system was also confirmed by traditional microbial culture methods using the BBL crystal enteric NF system (Becton Dickinson). Neither *P. aeruginosa* nor *S. marce*sc*ens* was detected in the HPW using the developed NAD test or by traditional microbial culture methods.

This current study, which was designed in accordance with MIQE guidelines [28], has focused on the development of a rapid methodology for the absolute quantification, detection and identification of four pathogenic bacteria from an operating HPW distribution system. Other studies have demonstrated that biocontamination of water and HPW distribution systems can occur with a number of other important human pathogenic microorganisms which were not investigated in this study [24,43,54]. We propose that with the inclusion of additional NAD tests, this methodology could be used to detect and identify a more comprehensive panel of pathogenic microorganisms (bacterial and fungal) for use in the analysis of microbial contaminants of water and HPW distribution systems. While the developed rapid culture independent methodology was demonstrated using a limited number of samples from a HPW distribution system the same methodology could be applied to other water and HPW distribution systems such as those in a clinical setting. We intend to evaluate a number of other water and HPW distribution systems over a longer period to further demonstrate the robustness of the developed methodology.

## Conclusion

As water and HPW is routinely used for a wide range of applications in the clinical and industrial sectors, it is essential that water purification units and water distribution systems are subject to strict maintenance schedules and stringent microbiological analysis. The rapid culture independent methodology described here may be a valuable diagnostics tool and have many useful applications in both these sectors.

## Additional file

Additional file 1: Table S1. Bacterial and fungal species and strains used in this study. **Table S2.** TVC from microbiological analysis of HPW delivery system by conventional culture methodologies.

## Abbreviations

HPW: High purity water
NAD: Nucleic acid diagnostics
PCR: Polymerase chain reaction
TAT: Turnaround time
HCAIs: Healthcare Associated Infections
GE: Genome equivalents
CFU: Colony forming units
TVC: Total viable counts
LOD: Limit of detection
HPCs: Heterotrophic Plate Counts (HPCs)
