# Supplementary material for: A rapid culture independent methodology to quantitatively detect and identify common human bacterial pathogens associated with contaminated high purity water
Source: BMC Biotechnol. 2015 Feb 18;15(1):6. doi: 10.1186/s12896-015-0124-1 (PMC4342816; doi:10.1186/s12896-015-0124-1)
Supplement: Additional file 1: Table S1. — Bacterial and fungal species and strains used in this study. Table S2. TVC from microbiological analysis of HPW delivery system by conventional culture methodologies. [file 12896_2015_124_MOESM1_ESM.docx]

**Table S1.** Bacterial and fungal species and strains used in this study

| **Species** | **Strain designation ^a,b,c^** | **Origin** |
| --- | --- | --- |
| *Pseudomonas aeruginosa* | DSM 50071^T^ | NA |
| *P. aeruginosa* PA01 | DSM 19880 | Human |
| *P. aeruginosa* | DSM 1117 | Human |
| *P. aeruginosa* | DSM 288 | NA |
| *P. aeruginosa* | DSM 1253 | NA |
| *P. aeruginosa* | DSM 1299 | Human |
| *P. aeruginosa* | DSM 1707 | NA |
| *P. aeruginosa* | DSM 3227 | Human |
| *P. aeruginosa* | DSM 11148 | NA |
| *P. aeruginosa* | DSM 46349 | Human |
| *P. aeruginosa* | DSM 1128 | Human |
| *P. aeruginosa* | DSM 24599 | Human |
| *P. aeruginosa* | DSM 46358 | Human |
| *P. aeruginosa* | DSM 50073 | NA |
| *P. putida* | DSM 291 ^T^ | NA |
| *P. alcaligenes* | LMG 1224 ^T^ | Environmental |
| *P. chlororaphis* | LMG 5004 ^T^ | Environmental |
| *P. mendocina* | LMG 1223 ^T^ | Environmental |
| *P. flavescens* | LMG 18387 ^T^ | Plant |
| *P. resinovorans* | LMG 2274 ^T^ | Environmental |
| *P. syringae* | LMG 1247 ^T (PVRS)^ | Plant |
| *P. fluorescens* | LMG 1794 ^T^ | Environmental |
| *P. nitroreducens* | LMG 21614 ^T^ | Environmental |
| *P. oleovorans* | LMG 2229 ^T^ | Environmental |
| *P. stutzerii* | LMG 11199 ^T^ | Human |
| *P. pertucinogena* | LMG 1874 ^T^ | Human |
| *Burkholderia cepacia* | DSM 7288 ^T^ | Plant |
| *B. contaminans* | DSM 22706 ^T^ | Animal |
| *B. diffusa* | DSM 23434 ^T^ | Human |
| *B. endofungorum* | DSM 19003 ^T^ | Fungal |
| *B. ferrariae* | LMG 23612 ^T^ | Environmental |
| *B. rhizoxinica* | DSM 19002 ^T^ | Fungal |
| *B. seminalis* | DSM 23518 ^T^ | Human |
| *B. sordidicola* | LMG 22029 ^T^ | Plant |
| *B. stabilis* | DSM 16586 ^T^ | Human |
| *B. tropica* | LMG 22274 ^T^ | Plant |
| *Serratia marcescens* | DSM 1608 | NA |
| *S. marcescens* subsp. *marcescens* | DSM 30121 ^T^ | Environmental |
| *S. marcescens* | DSM 30126 | Environmental |
| *S. marcescens* | DSM 1636 | NA |
| *S. marcescens* subsp. ***sakuensis*** | DSM 17174 ^T^ | Environmental |
| *S. symbiotica* | DSM 23270 ^T^ | Plant |
| *S. entomophila* | DSM 12358 ^T^ | Animal |
| *S. fonticola* | DSM 4576 ^T^ | Environmental |
| *S. rubidae* | DSM 4480 ^T^ | NA |
| *S. odoriferae* | DSM 4582 ^T^ | Human |
| *Stenotrophomonas maltophilia* | DSM 50170 ^T^ | Human |
| *S. maltophilia* | DSM 21874 | Environmental |
| *S .maltophilia* | DSM 50173 | Environmental |
| *S. maltophilia* | DSM 30740 | Environmental |
| *S. maltophilia* | DSM 24970 | Human |
| *S. koreensis* | DSM 17805 ^T^ | Environmental |
| *S. chelatiphaga* | DSM 21508 ^T^ | Environmental |
| *S. acidiminiphila* | LMG 22073 ^T^ | Environmental |
| *S. nitroreducens* | LMG 22074 ^T^ | Environmental |
| *S. rhizophila* | LMG 24537 ^T^ | Plant |
| Xanthomonas *albilineans* | LMG 494 ^T^ | Plant |
| *X. axonopodis* | LMG 982 ^T (PVRS)^ | Plant |
| *X. campestris* | DSM 3586 ^T^ | Plant |
| *Sphingomonas paucimobilis* | DSM 1098 ^T^ | Environmental |
| *S. adhaesiva* | LMG 10922 ^T^ | Environmental |
| *S. parapaucimobilis* | DSM 7463 ^T^ | Human |
| *S. paucimobilis* | LMG 2239 | Human |
| *Ralstonia sp.* | DSM 13640 | Environmental |
| *R. pickettii* | DSM 6297 ^T^ | NA |
| *R. syzygii* | DSM 7385 ^T^ | Plant |
| *R. mannitolilytica* | LMG 18098 | Human |
| *R. insidiosa* | LMG 18111 | Human |
| *Cupriavidus basilensis* | DSM 11853 ^T^ | Environmental |
| *C. pauculus* | DSM 17313 ^T^ | Human |
| *C. gilardii* | DSM 17292 ^T^ | Environmental |
| *Candida metapsilosis* | CBS 2315 | Human |
| *C. krusei* | DSM 70075 | Human |
| *Bacillus cereus* | DSM 31 ^T^ | NA |
| *B. cereus* | DSM 508 | NA |
| *B. mycoides* | DSM 2048 ^T^ | Environmental |
| *B. subtilis* subsp. ***inaquosorum*** | DSM 22148 ^T^ | Environmental |
| *C lostridium difficile* | DSM 1296 ^T^ | NA |
| *C. difficle* | DSM 12056 | Animal |
| *Klebsiella pneumoniae* subsp. ***pneumoniae*** | DSM 9377 | Human |
| *K. oxytoca* | DSM 5175 ^T^ | Human |
| *Raoultella terrigena* | DSM 7331 | Human |
| *Proteus mirabilis* | DSM 4479 ^T^ | NA |
| *P. vulgaris* | DSM 2140 | Human |
| *Staphylococcus epidermidis* | DSM 20044 ^T^ | Human |
| *S. aureus* | DSM 2569 | Human |
| *S. aureus* | DSM 21705 | Human |
| *Enterobacter aerogenes* | LMG 26130 | Human |
| *E. aerogenes* | NCTC 10006 ^T^ | Human |
| *E. cloacae* | ATCC 13047 ^T^ | Human |
| *E. asburiae* | DSM 17506 ^T^ | Human |
| *Acinetobacter iwoffii* | DSM 2403 ^T^ | NA |
| *A. calaoaceticus* | LMG 1046 ^T^ | Environmental |
| *A. baumannii* | DSM 30007 ^T^ | Human |
| *Haemophilus influenzae* | DSM 24049 ^T^ | Human |

# ^a^*DSM = Deutsche Sammlung von Mikroorganismen und Zellkulturen GmbH, The German Collection of Microorganisms; * LMG=Laboratorium voor Microbiologie -Belgian Co-ordinated Collections of Micro-organisms; * CBS=Centraalbureau voor Schimmelcultures, Fungal Biodiversity Centre, Netherlands; *ATCC= American Type Culture Collection; * NCTC= National Collection of Type Cultures, a Culture Collection of Public Health England.

^b^PVRS= Pathovar reference strain

T = Type strain

**Table S2.** TVC from microbiological analysis of HPW delivery system by conventional culture methodologies

| Sampling point | Sampling time | Mean calculated cfu / 100 ml from TVC (SD) |
| --- | --- | --- |
| A | 1 | 3.42x10^5^ ( 245) |
| A | 2 | 3.54 x10^5^ (268) |
| A | 3 | 2.63 x10^5^ (124) |
| B | 1 | 3.67 x10^5^ (279) |
| B | 2 | 3.27 x10^5^ (197) |
| B | 3 | 2.93 x10^5^ (169) |

A Valve adjacent to Elix 35 HPW purification system

B Laboratory tap approximately 25 - 30 m from the Elix 35 HPW purification system

1 Before Pretreatment and Polishing Pack change

2 one day post Pretreatment and Polishing Pack change

3 one week post Pretreatment and Polishing Pack change
